# Acceptable outcome following resection of bilateral large popliteal space heterotopic ossification masses in a spinal cord injured patient: a case report

**DOI:** 10.1186/1749-799X-5-39

**Published:** 2010-06-22

**Authors:** Ramin Espandar, Babak Haghpanah

**Affiliations:** 1 Department of orthopaedic surgery, Imam Khomeini Hospital Complex, Tehran University of Medical Sciences, Keshavarz Blvd, Tehran 1419733141, Iran

## Abstract

Spinal cord injury is a well-known predisposing factor for development of heterotopic ossification around the joints especially hip and elbow. Heterotopic ossification about the knee is usually located medially, laterally or anteriorly; besides, the knee is generally fixed in flexion. There are only a few reports of heterotopic bone formation at the posterior aspect of the knee (popliteal space) and fixation of both knees in extension; so, there is little experience in operative management of such a problem.

Here, we present a 39-years old paraplegic man who was referred to us five years after trauma with a request of above knee amputation due to sever impairment of his life style and adaptive capacity for daily living because of difficulties in using wheelchair. The principle reason for the impairment was fixed full extension of both knees as the result of bilateral large heterotopic ossification masses in popliteal fossae. The bony masses were surgically resected with acceptable outcome. The anatomic position of the ossified masses as well as ankylosis of both knees in full extension, and the acceptable functional outcome of surgery which was done after a long period of five years following injury makes this case unique.

## Background

Heterotopic ossification (HO) is formation of lamellar bone within the soft tissue structures. During its course of evolution, HO turns into mature bone structure with cortex and medullary cavity containing bone marrow cells and variable amount of hematopoiesis. The exact mechanism by which such a process begins and evolves is not clearly understood but various hypotheses are proposed. Formation of heterotopic bone is known to be associated with some predisposing etiologies such as neurogenic, traumatic, genetic and some surgical procedures [1]. Because HO most commonly involves the large joints [2], significant morbidity and functional deficit may result regardless of the primary etiology [3-6]. Established lesions of HO which interfere with function, ambulation or posture, predispose to pressure sores, or cause intractable pain are amenable to surgical resection. Surgical resection of heterotopic bone results in significant improvement of functional state of the patients [6-10]. We report a case of bilateral popliteal fossa HO and paraplegia due to spinal cord injury five years before, and the resultant fixed knee ankylosis in full extension. He referred to us with complain of difficulty using wheelchair and problem with ambulation both indoors and outdoors. To our knowledge there is no report of surgical management and functional outcome after excision of HO in posterior knee to facilitate ambulation of paraplegic patient after this long period of time following injury.

## Case Presentation

A 39-years-old man was referred to our clinic with complain of inability to sit on and use wheelchair for ambulation due to the lack of flexion in both knees. He was a victim of a diving accident 5 years before presentation after which he had been quadriplegic. With appropriate care and surgical intervention upper extremities regained some function (sensory and motor) but the paraplegia remained. During the first 6 months following the injury he noticed progressive lack of flexion of both knees and finally total ankylosis of both knees in full extension. The problem severely impacted his lifestyle and mobility due to impaired sitting ability. The problem bothered the patient so that he would request an amputation if the position of the knee joint could not be corrected.

**Figure 1:**
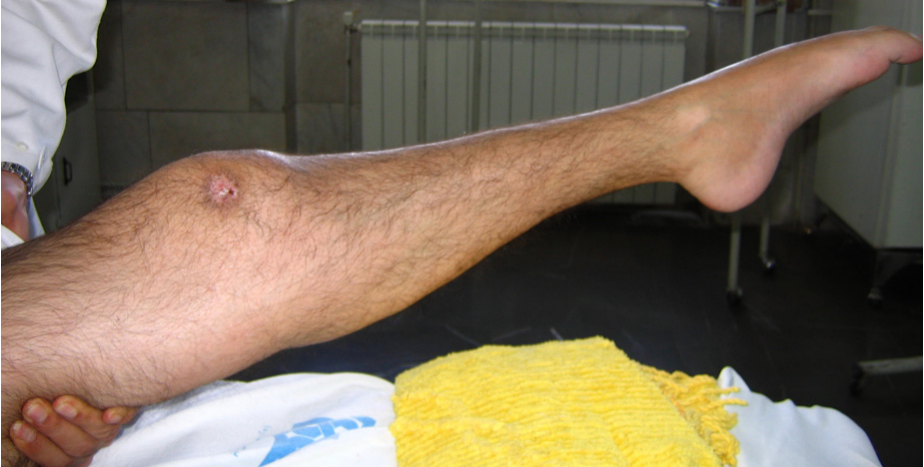
**Physical examination of the patient, the knee is stiff in extension and ankle is fixed in equinus position**.

On physical examination, the knees had no passive movement and both ankles were fixed in equinus position (Figure 1). A burn scar was seen on the lateral aspect of the right knee. Distal posterior tibialis and dorsalis pedis pulses were palpated and were symmetric. On neurologic examination there were no voluntary contraction in his spastic lower limbs and complete sensory deficit was evident. The patient was under treatment with warfarin due to previous deep vein thrombosis. The medication was changed to heparin before operation.

**Figure 2:**
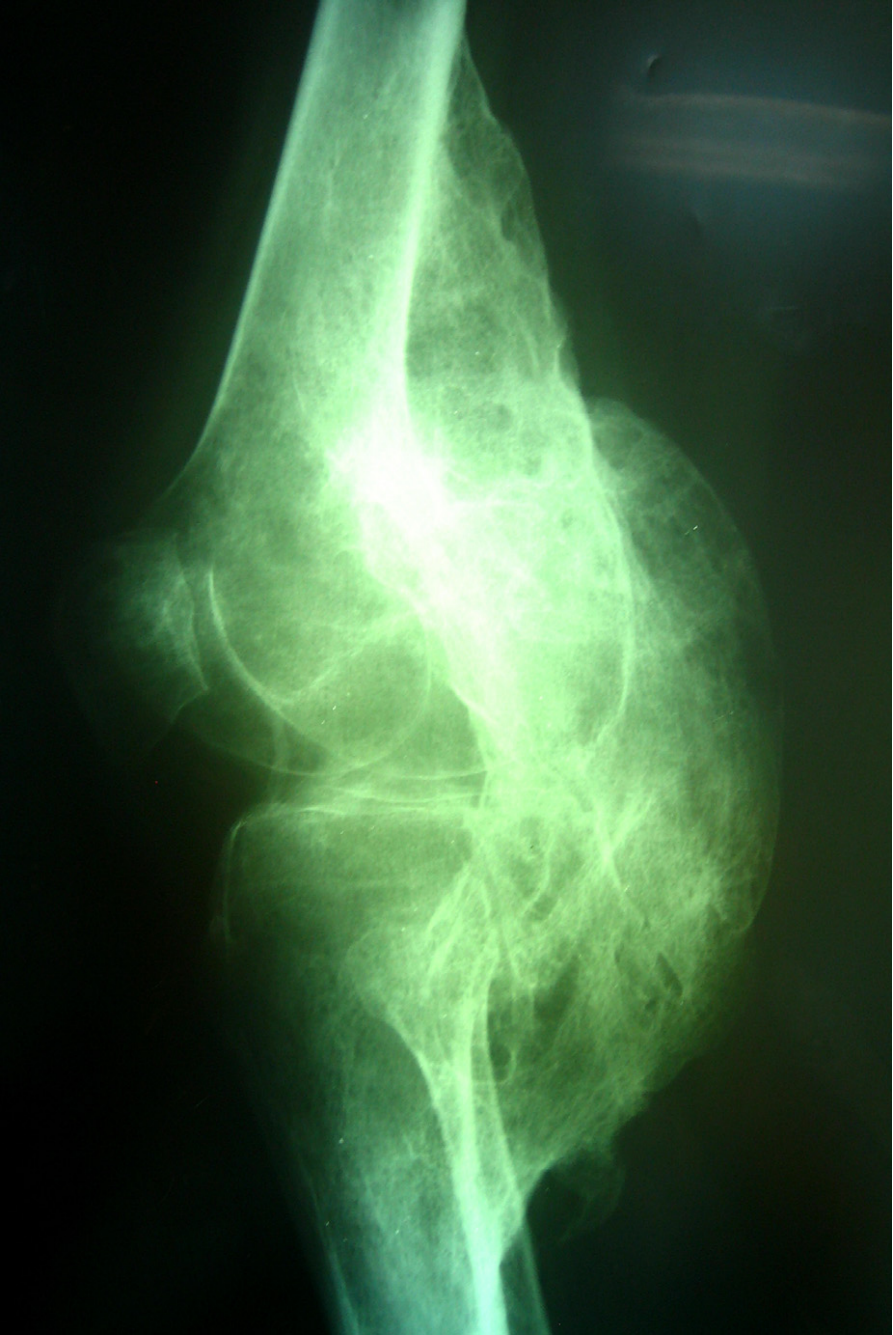
**Lateral radiography of the right knee, large mass of heterotopic bone is seen bridging the knee joint posteriorly**.

**Figure 3:**
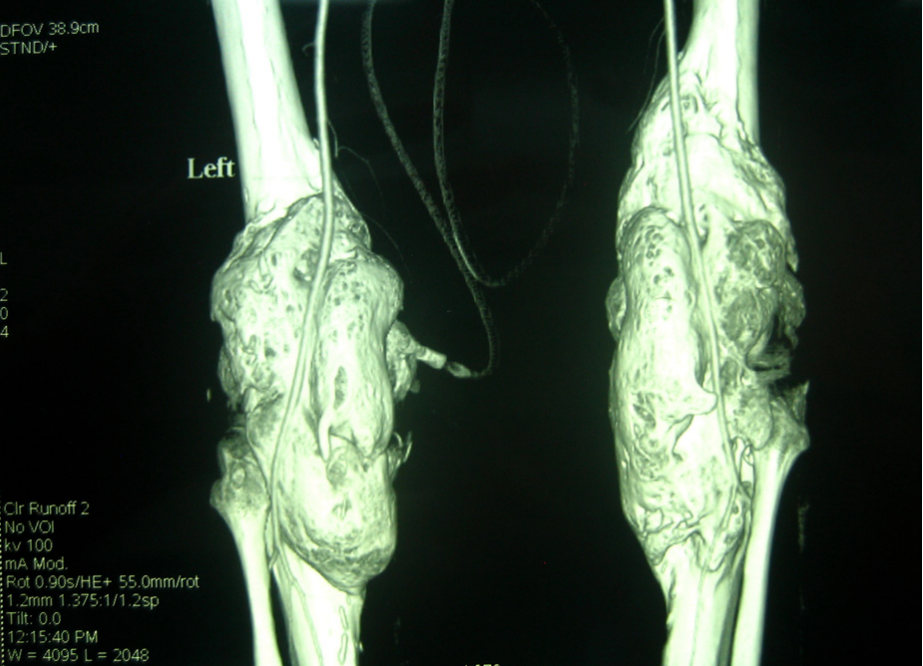
CT Angiography of the knees; both popliteal arteries encased in grooves of the heterotopic bone.

On radiographic examinations, large masses of heterotopic bone were seen bridging the knee joints from posterior distal femur to proximal tibia in the popliteal fossa (Figure 2). To determine the vicinity of neurovascular structures with the heterotopic bone a CT-angiography was performed which showed both popliteal arteries displaced posteriorly and encased in grooves of heterotopic bone (Figure 3). On the right side the mass was larger (220 × 50 mm) starting more proximally from about the Hunter's canal down distal to the level of trifurcation of popliteal artery. On left side the mass (180 × 65 mm) ended just proximal to the level of trifurcation. An MRI study was done to assess the integrity of articular structures and to rule out the articular involvement.

On laboratory data, erythrocyte sedimentation rate (ESR) and C-reactive protein (CRP) and alkaline phosphatase (ALP) levels were normal. Bone scintigraphy was not performed.

**Figure 4:**
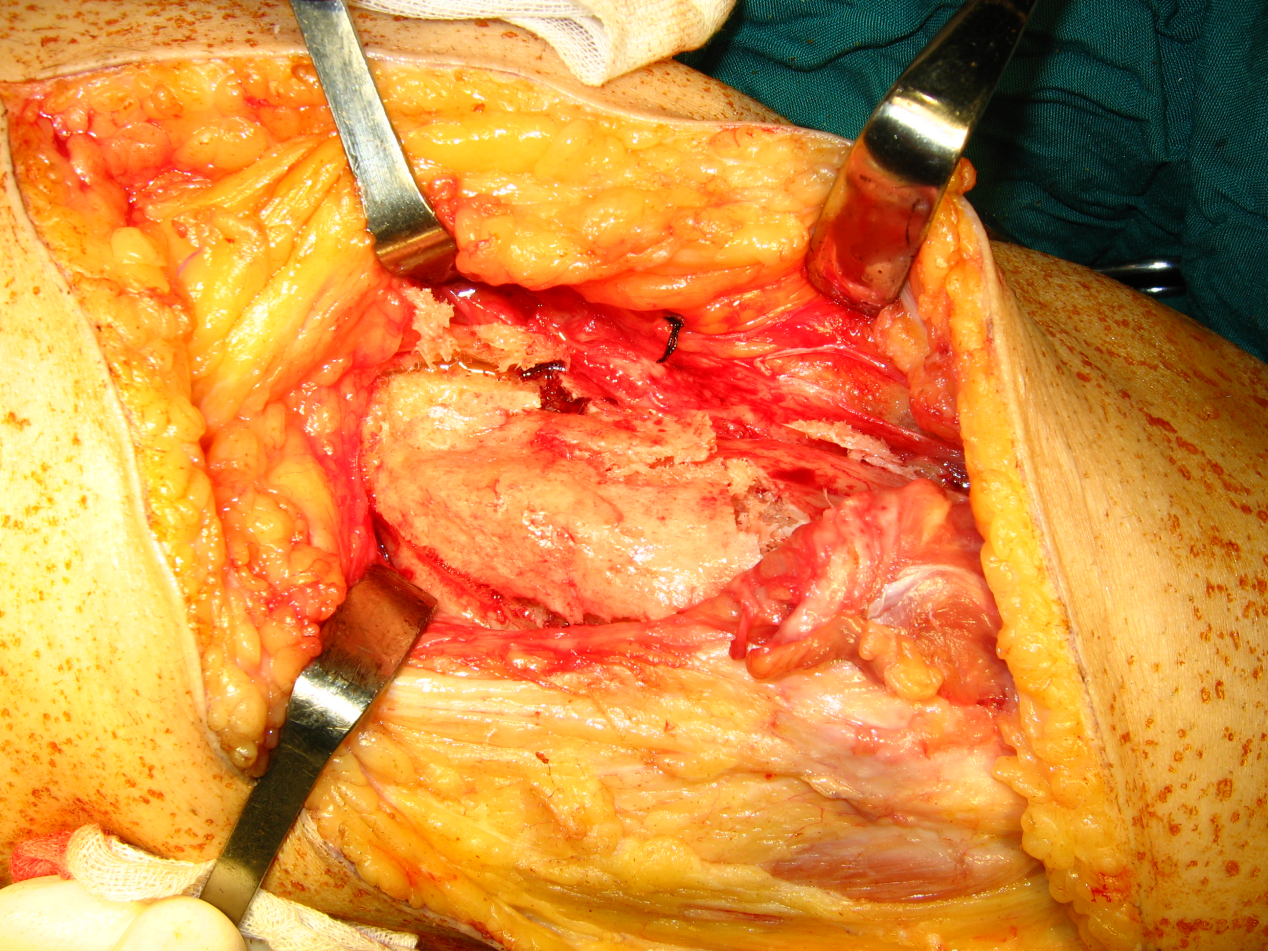
**Gastrocnemius and the hamstrings were not involved in the heterotopic bone mass**.

Vascular surgical consultation was requested regarding the vicinity of popliteal vessels to the mass, and the risks of surgery was discussed with the patient. The left knee was operated on first due to presence of fresh scar and ulceration on lateral side of the right knee. With the patient in prone position and under general anaesthesia, posterior approach with lazy-S incision was used. Medial head of the gastrocnemius muscle was released. The gastrocnemius and the hamstrings were atrophic but not involved in the heterotopic bone (Figure 4). After ligation of the superior medial genicular branch, the popiteal artery was explored and dissected free in its entire length. The mass was excised using osteotome in its base. The posterior knee capsule was involved in the mass and was resected partially. Posterior cruciate ligament was seen intact. We gained 0 to 95 degrees of flexion intraoperatively. The tourniquet was deflated and hemostasis done. Posterior tibialis and dorsalis pedis pulses were checked. Suction drain was placed and wound closed in usual manner. A hinged knee brace was placed locked in 60 degrees flexion.

Postoperative prophylaxis was done with a single dose administration of 700cGy irradiation on the first day. Indomethacin was given 75 mg daily and continued for 6 weeks. Prophylactic administration of Enoxaparin 40 mg daily (for deep vein thrombosis) started on first postoperative day. The drains were removed on second postoperative day and the brace unlocked to start full gentle range of motion. On the fourth postoperative day the patient developed serousanguinous discharge from the wound which resolved after 2 days. On the third postoperative week the patient referred with a pitting edema of the left foot. Color doppler ultrasonography revealed deep vein thrombosis of calf which mandated medical treatment of the thrombosis. The postoperative course was otherwise uneventful. Pathologic study was compatible with heterotopic ossification. On sixth postoperative month the range of motion was 0 to 80 degrees of flexion. The right knee was operated 3 months after the left one with the same surgical technique and the same surgeon. Immediate postoperative range of motion was 0 to 100 degrees of flexion. The postoperative follow up was the same as the left one with no complications. After sixth months, the range of motion of right knee was 0 to 75 degrees of flexion.

## Discussion

There are few reports of posterior knee HO in the literature. In a study by Garland et al. [11] three cases of HO of the knee were reported. In only one of them the lesion was located in the posterior knee and none of the cases developed ankylosis of the knee. In the series published by Charnley et al. [7] and Ippolito et al. [12] no cases of popliteal space HO were reported. To our knowledge, there are rare reports of excision of large popliteal space HO. In a report by Anderson and Lais [13] a large HO mass was excised from popliteal fossa of a 20 years old man 17 weeks after traumatic brain injury. The patient had a fixed flexion contracture of 45 degrees. At 7 months follow-up the patient had a range of motion of 10-125 degrees. They used both irradiation therapy and indomethacin for postoperative prophylaxis. We found no reports of popliteal space HO except the aforementioned study. Our patient is unique in that his knees were fixed in full extension and that surgical intervention for resection of the lesion was done 5 years after development of the HO. We attribute the postoperative residual flexion deficit at least partly to the contracture of extensor mechanism in extension during the long period of time.

Spinal cord injury is a well known predisposing factor for development of HO. The incidence of HO after spinal cord injury has been reported to be 20-25% [11]. The most common joints involved are hip, shoulder, elbow and the knee in order of decreasing frequency[14]. Involvement of knee joint with HO has marked effect on functional status of the patients significantly reducing their adaptive capacity for daily living [6,7,9]. Fuller et al. [6] reviewed 17 patients with 22 knees involved by heterotopic ossification and categorized their sitting impairment and investigated their functional outcome after resection of the lesions. He classified the patients as: group I (patients who are able to use a wheelchair or a chair without being assisted), group II (patients who can use chair only with the help of assistive devices such as cushions or chair extensions) and group III (patients who are not able to use chair even with assistance).

Multiple researchers have shown the benefit of surgical excision of HO lesions of the knee in overall functional status of the patients [6,9,10]. Traditionally, the optimal time for resection of heterotopic ossification was considered to be after maturation of the lesion (normalization of bone scan). This was thought to reduce the recurrence of the lesion. Recently, earlier surgical intervention has been recommended by some authors. Melamed et al. reported excision of 12 HO lesions in 9 patients [15]. Despite increased uptake on bone scans in all patients, recurrence did not occur in any of them. They suggested that increased uptake on bone scans is not a contraindication to surgical excision of HO, provided the neurologic status is stabilized. Importance of neurological status of the patients and its impact on the results of surgery has been emphasized by other authors. Sarafis et al. [16] attributed the poor functional outcome of their patients after excision of HO in 22 hips to their uncontrolled neurologic syndrome. They recommended accurate evaluation of the preoperative neurologic status. On the other hand, they warned about the risk of fracture in delayed surgery due to localized osteoporosis. Delay in surgical intervention may also have a detrimental effect on regaining the range of joint motion, adversely influencing the efficacy of rehabilitation programs.

The exact etiology and pathophysiology of HO is not clearly defined. Chalmers et al. studied the inducing capacity of different tissues for bone formation [17]. He believed the presence of three conditions is necessary for development of ossification within soft tissues: 1) an inducing agent; 2) an osteogenic precursor cell; and 3) an environment which is permissive to osteogenesis. A large amount of information regarding the pathophysiology of HO has been collected by studying the cases of myositis ossificans progressiva; an inherited disorder with progressive debilitating ossification of soft tissue structures [18-23]. The role of bone morphogenic proteins (BMPs) and its antagonists such as noggin has recently been the focus of attention. It is postulated that the BMP-4 gene itself may not be defective but a defect in the genes that code BMP-4 antagonists leads to suppression of inhibitory mechanisms and overexpression of BMP-4 [24].

Recurrence of HO after surgical resection is one of the most common complications affecting the final outcome. The role of prostaglandine E2 (PGE2) in pathophysiology of HO and its increased urinary excretion in early stages of the disease has been the rational for use of non steroidal anti-inflammatory drugs (NSAIDs) as a preventive measure. Indomethacin has been of particular interest. Indomethacin appears to be effective in the primary prevention of HO after spinal cord injuries and after total hip arthroplasty and as secondary preventive measure after resection of HO lesions [25]. The major drawback of indomethacin use is the increased risk of operative bleeding, its gastrointestinal side effects and its negative effect in bone union. Other more selective NSAIDs have been studied for this reason and their efficacy and safety is under investigation. Radiation therapy has been used extensively for the prevention of HO. Many side effects seen with the use of indomethacin are not the concern with irradiation. With proper shielding, irradiation can be applied to only where it is needed. However, despite the low doses used for HO prophylaxis, the risk of carcinogenesis is a concern. Most articles about the effects of radiation therapy in prevention of HO focus in post-total hip arthroplasty (THA) cases. The studies about the preventive effects of radiation therapy are plagued with small sample sizes and inadequate research protocol design. The optimal dose and fractionation of dosage are subjects of some researches [26].

Popliteal space HO is a rare affliction. With presentation of our case, we believe that by resection of popliteal space knee HO, good function and improvement of life style can be anticipated even after a long delay in presentation. Appropriate postoperative prophylaxis with radiotherapy and NSAIDs should be considered in treatment course.

## Consent

Written informed consent was obtained from the patient for publication of this case report and accompanying images. A copy of the written consent is available for review by the Editor-in-Chief of this journal.

## Competing interests

The authors declare that they have no competing interests.

## Authors' contributions

RE was the senior surgeon who performed the surgical procedure, helped with the concept and revised the manuscript.

BH participated in surgery and follow-up and drafted the manuscript. All authors read and approved the final manuscript.
